# Potential and Challenges of Induced Pluripotent Stem Cells in Liver Diseases Treatment

**DOI:** 10.3390/jcm3030997

**Published:** 2014-09-05

**Authors:** Yue Yu, Xuehao Wang, Scott L. Nyberg

**Affiliations:** 1 Key Laboratory of Living Donor Liver Transplantation, Ministry of Public Health, Nanjing, Jiangsu Province 210029, China; E-Mails: yuyue@njmu.edu.cn (Y.Y.); wangxh@njmu.edu.cn (X.W.); 2 Liver Transplantation Center, The First Affiliated Hospital of Nanjing Medical University, Nanjing, Jiangsu Province 210029, China; 3 Division of Experimental Surgery, Mayo Clinic College of Medicine, Rochester, MN 55905, USA

**Keywords:** iPSCs, liver disease, animal model, hepatic differentiation

## Abstract

Tens of millions of patients are affected by liver disease worldwide. Many of these patients can benefit from cell therapy involving living metabolically active cells, either by treatment of their liver disease, or by prevention of their disease phenotype. Cell therapies, including hepatocyte transplantation and bioartificial liver (BAL) devices, have been proposed as therapeutic alternatives to the shortage of transplantable livers. Both BAL and hepatocyte transplantation are cellular therapies that avoid use of a whole liver. Hepatocytes are also widely used in drug screening and liver disease modelling. However, the demand for human hepatocytes, heavily outweighs their availability by conventional means. Induced pluripotent stem cells (iPSCs) technology brings together the potential benefits of embryonic stem cells (ESCs) (*i.e*., self-renewal, pluripotency) and addresses the major ethical and scientific concerns of ESCs: embryo destruction and immune-incompatibility. It has been shown that hepatocyte-like cells (HLCs) can be generated from iPSCs. Furthermore, human iPSCs (hiPSCs) can provide an unlimited source of human hepatocytes and hold great promise for applications in regenerative medicine, drug screening and liver diseases modelling. Despite steady progress, there are still several major obstacles that need to be overcome before iPSCs will reach the bedside. This review will focus on the current state of efforts to derive hiPSCs for potential use in modelling and treatment of liver disease.

## 1. Introduction

Tens of millions of patients are affected by liver disease worldwide. Liver transplantation, the ultimate cell therapy, is presently the only proven treatment for many medically refractory liver diseases including end-stage liver disease and many inherited liver diseases. However, there is a profound shortage of transplantable donor livers. Regenerative medicine, which focuses on innovative approaches to repairing and replacing cells, tissues and organs, is undergoing significant revolution do to the unprecedented world-wide demand for organs. Many of the liver disease patients can benefit from cell therapy involving metabolically active cells. Cell therapies, including hepatocyte transplantation and bioartificial liver (BAL) devices, have been proposed as therapeutic alternatives to the shortage of transplantable livers. BAL is an extracorporeal supportive therapy developed to bridge patients with liver failure to liver transplantation or to recovery of the native liver. Hepatocyte transplantation is best suited for patients with metabolic liver disease for which smaller number of cells (<10% of liver mass) may be curative. Both BAL and hepatocyte transplantation are cellular therapies that avoid use of a whole liver. Hepatocytes are also widely used in drug screening and liver disease modelling. However, conventional methods of obtaining hepatocytes cannot meet clinical demand because of the shortage of donor livers from which high quality hepatocytes can be isolated. Furthermore, hepatocytes are not easily maintained in culture over extended periods of time. Moreover, hepatocyte propagation is minimal *in vitro*, even in the presence of growth factors such as hepatocyte growth factor [1]. Hepatocytes are also difficult to cryopreserve and highly susceptible to freeze-thaw damage [2]. The demand for human hepatocytes, therefore, heavily outweighs their availability. 

The recent discovery that human induced pluripotent stem cells (hiPSCs) can be derived from human somatic cells through forced expression of defined transcription factors such as OCT4 (O), SOX2 (S), KLF4 (K), and c-MYC (M) (so called OSKM cocktail) or O, S, NANOG (N) and LIN28 (L) (so called OSNL), has renewed hopes for regenerative medicine and *in vitro* disease modelling, as these cells are easily accessible. iPSC technology brings together the potential benefits of embryonic stem cells (ESCs) (*i.e*., self-renewal, pluripotency) and addresses major ethical and scientific concerns of ESCs: (1) iPSCs bypass the ethical concerns of embryo destruction since they are produced from somatic cells *in vitro* without embryonic tissues or oocytes; (2) the immune-compatibility issues since they are generated from patient-specific cell types. The field of iPSCs has undergone tremendous growth, and differentiated cell types produced from a patient’s iPSCs have demonstrated many potential therapeutic applications, including their use in tissue replacement and gene therapy. It was shown that HLCs could be generated from iPSCs. Our previous study [3] and others’ reports [4] of the potential benefits of HLCs generated from hiPSCs have described their secretion of human albumin, alpha-1-antitrypsin (A1AT), and hepatocyte nuclear factor 4-alpha (HNF4α), synthesis of urea, and expression of cytochrome P450 (CYP) enzymes *in vitro*. Therefore, in theory, hiPSCs could provide an unlimited source of human hepatocytes and hold great promise for applications in regenerative medicine, drug screening and liver diseases modelling. More recently, investigators have reported that HLCs differentiated from hiPSCs of patients with the inherited metabolic conditions may be used to model inherited liver diseases [5]. Transplantation of HLCs derived from hiPSCs may provide alternatives to liver transplantation for the treatment of acute liver failure (ALF), liver cirrhosis, viral hepatitis, and the correction of inherited metabolic liver disorders. This review will focus on the current state of efforts to derive hiPSCs for potential use in modelling and treatment of liver disease.

## 2. Hepatic Differentiation of iPSCs *in Vitro*

We will first address the hepatic differentiation of iPSCs. The term HLCs refers to cells produced *in vitro* that possess some of the properties of mature hepatocytes. Song *et al*. [6] first demonstrated that hiPSCs can be induced to HLCs directly by the administration of various growth factors in a time dependent manner. The sequence of differentiation follows the normal sequence of human liver development, and includes: Stage 1-endoderm induction, Stage 2-hepatic specification, Stage 3-hepatoblast expansion and Stage 4-hepatic maturation. Subsequent reports have focused on optimizing this method by adding modifications and improvements to the differentiation protocols. In addition to growth factors, a variety of factors have been used to enhance the differentiation of hiPSCs towards the hepatic lineage. For example, small molecules (*i.e*., dimethyl sulfoxide (DMSO), dexamethasone) have been shown to extend the hepatic differentiation of iPSCs. The characteristics of pluripotent stem cell derived HLCs produced from various differentiation protocols have been critically reviewed [7]. Zhang *et al*. induced efficient generation of highly differentiated HLCs from mouse iPSCs by a combination of cytokines and sodium butyrate [8]. To promote hepatic maturation, Takayama utilized transduction of the hepatocyte HNF4α gene, which is known as a master regulator of liver-specific gene expression. Over expression of HNF4α in hepatoblasts derived from iPSCs led to up-regulation of markers of epithelial and mature hepatic development, such as CYP enzymes. HNF4α also promoted hepatic maturation by activating the mesenchymal-to-epithelial transition. The Takayama method is also a valuable tool for the efficient generation of functional hepatocytes derived from human ESCs and iPSCs, and these HLCs have been used for predicting drug toxicity [9]. A limitation of these four-stage protocols is that they are time consuming, usually requiring more than 20 days. In contrast, Chen [10] reported rapid generation of HLCs from hiPSCs by an efficient three-step protocol. Using Chen’s system, the differentiation of hiPSCs into functional HLCs requires only 12 days. Chen’s method is different from the typical protocols as they apply HGF in the first stage (endoderm induction), rather than during the hepatocyte maturation stage. It is expected that future research will facilitate the differentiated of iPSCs to fully mature functional hepatocyte.

## 3. Applications of iPSCs in Liver Diseases

The applications of iPSCs in liver diseases will be outlined in the Table 1.

### 3.1. Regenerative Medicine

iPSCs-Derived HLCs from normal individuals can be used in the establishment of cell banks for applications in regenerative medicine. Results from various studies have demonstrated the therapeutic potential of iPSCs-derived HLCs in liver diseases. Examples of the therapeutic potential of iPSC-derived HLC’s in rodent models include *vivo* transplantation of HLCs to reverse lethal fulminant hepatic failure [10], both the functional and proliferative potential of HLCs for enhanced liver regeneration [11], reduced liver fibrosis [12], and stabilization of chronic liver disease [13]. Disease models have utilized immunodeficient mice and immunosuppression to demonstrate a therapeutic benefit of human HLC’s. For human application, generating hiPSC-derived HLCs from selected adults and construction of libraries of cell lines with known genotypes, providing patients with a close HLA/MHC match, may minimize the need for immunosuppression to achieve cell engraftment. hiPSCs also introduce the possibility of patient-derived HLCs which will be discussed later.

**Table 1:** Applications of iPSCs in liver diseases.

Applications	Diseases	iPSCs from Donor	References
Regenerative Medicine	fulminant hepatic failure	mouse normal iPSCs	[10]
	liver regeneration	mouse normal iPSCs	[11]
	liver fibrosis	mouse normal iPSCs	[12]
	chronic liver disease	mouse normal iPSCs	[13]
BAL	liver failure	human normal iPSCs	[14]
Gene Therapy	A1AT deficiency	A1AT deficiency patient iPSCs	[15]
	WD	WD patient	[16]
Liver Diseases Model	HBV, HCV	human normal iPSCs	[17]
	HCV	human normal iPSCs	[18,19]
	A1AT deficiency GSD, FH, Crigler-Najjar syndrome, hereditary tyrosinemia	patients iPSCs	[5,20]
Drug Discovery and Hepatoxicity Screening	Normal liver	patients iPSCs or human normal iPSCs	[5,21,22,23,24,25]

### 3.2. BAL

The incidence of ALF is approximately 2500 cases per year in the United States and is much higher worldwide [26]. The shortage of liver donor for transplantation leads to approximately 40% of listed patients per year not receiving a liver transplant with a significant number of these patients either dying or becoming too sick to transplant. BAL is an extracorporeal supportive therapy developed to bridge patients with liver failure to liver transplantation or to recovery of the native liver. The BAL system removes toxins by filtration or adsorption (artificial liver) while performing biotransformation and synthetic functions of biochemically active hepatocytes. A major question in the clinical application of liver support devices is how to supply them with adequate numbers of functional hepatocytes to improve patient survival. Fortunately, cells in the BAL are separated from the patient’s circulation by a semi-permeable membrane to prevent allogenic rejection, thus patient-specific hepatocytes are not needed.

To date, the various cell types that have been used in BAL devices have included primary human hepatocytes, primary porcine hepatocytes, immortalized human cell lines, fetal liver cells, and stem cell-derived cells. Primary human hepatocytes are not available in sufficient amounts needed for clinical usage of BAL, exceeding 200 grams per treatment. Furthermore, primary hepatocytes are limited by the short duration that they retain functionality and viability *in vitro*. Porcine hepatocytes are limited by immunogenic reactions resulting from the xenogenicity of porcine hepatocyte products and the possibility of xenozoonotic retroviral infection of patients with porcine endogenous retrovirus (PERV). However, the BAL membrane mitigates these concerns (the risk has never been quantified since no cases exist). Also of concern, immortalized cell lines lack essential functions, particularly the loss of urea cycle activity and lack of CYP enzyme expression [27]. Transfection methods to enable overexpression of CYP enzymes in these cells have been adopted, but this approach is limited by the expression of one CYP isoform per cell line and therefore does not fully recapitulate the metabolic capacity of a fully functional hepatocyte [28]. Alternatively, human ESCs and iPSCs differentiated HLCs show great promise as cell sources for BAL devices. Patient specific iPSCs-HLCs transplantation can provide effective treatment to liver diseases. Of concern in acute situations, such as ALF, time to make, mature, and expand the patient’s somatic cells into iPSCs and then HLCs may be prohibitive for treatment of ALF, either as a cell transplant or BAL therapy. The cell banks of normal individual-derived iPSCs with close HLA/MHC match to the ALF patient and then rapid differentiation into HLCs for use in BAL and temporary treatment of ALF deserves further investigation.

The Fox group has reported an implantable BAL device containing HLCs derived from ESCs in a murine model of liver failure [29]. They differentiated mouse ESCs into HLCs by coculture with a combination of human liver nonparenchymal cell lines and cytokines. Functional hepatocytes were isolated using albumin promoter-based cell sorting. The coculture differentiation strategy induced a 50% increase in the number of ESCs becoming albumin positive, and resulted in 68.7% of the entire cell population differentiating toward a hepatocyte phenotype. This may be due to the heterotopic interactions between hepatocytes and hepatic nonparenchymal cells in liver development. Treatment of 90% hepatectomized mice with a subcutaneously implanted BAL seeded with ESCs-derived HLCs improved liver function and prolonged survival. Iwamuro [14] tested a BAL system whose cell source was HLC’s derived from mouse iPSCs. These cells were injected into a hollow fiber module with a 0.2-μm pore size. The murine HLC’s adhered to the hollow fiber surface and produced albumin and urea for 7 days. Although further investigation and improvement of the device and the differentiation process are required, the authors concluded that the combination of a 0.2-μm pore membrane and iPSC-derived HLCs showed promise as an improved BAL system. This paper provides the basic concept and preliminary data for BAL as an individualized treatment system employing the patient’s own cells. Despite the paucity of reports addressing functionality of HLCs derived from hiPSCs in BAL systems, we believe hiPSCs-HLCs are a promising resource for BAL therapy.

### 3.3. Gene Therapy in Hereditary Liver Disease

The liver is affected by many types of diseases, including inherited metabolic disorders. A major indication for hepatocyte transplantation is inherited metabolic liver diseases in children. The liver is a vital organ that represents a promising target for cell therapy, because of its ability to functionally integrate transplanted hepatocytes. Hepatocyte transplantation has been performed as a treatment for inherited liver diseases, either for bridging to whole organ transplantation or for long-term correction of the underlying metabolic deficiency [30]. However, as mentioned earlier, the both shortage of donor organs from which to isolate high quality primary hepatocytes and the possibility of allogeneic rejection hamper the advance of hepatocyte transplantation. Patient-specific cell therapy is an ideal option to prevent cell rejection. However, isolation of autologous hepatocytes requires a lobectomy (resection of at least 20% of the liver), a procedure with risk in patients. Fortunately, development of iPSCs from patient somatic tissues and then differentiation into HLCs may provide patient specific hepatocyte source for treatment for inherited liver diseases. In the case of monogenic inherited metabolic liver diseases, in which all the cells from the body initially carry the disease-causing mutation in their genomic DNA, a gene correction approach is required to generate disease-free autologous cells. Thus, a combination of *ex vivo* gene therapy and cell transplantation has been considered [31,32].

iPSC-based gene/cell therapies have been applied in several animal models of liver-based metabolic disorders, with encouraging results. Yusa performed targeted gene correction of A1AT deficiency in iPSCs [15]. Mutation in A1AT gene is most commonly associated with Pizz-associated liver disease leading to cirrhosis. These investigators used the combined method of zinc finger nucleases (ZFNs) and piggyBac (PB) technology in hiPSCs to achieve biallelic correction of the culprit point mutation (Glu342Lys) in the A1AT gene. Genetic correction of hiPSCs restored the structure and function of HLC’s *in vitro* and subsequently corrected A1AT *in vivo*. Transplantation of these iPSC-derived HLCs into immunodeficient mice was able to produce albumin and provide functional A1AT protein. This approach is significantly more efficient than other gene-targeting technology currently available, and does not require homologous recombination that leaves residual sequences in the targeted genome, and which leads to unintended consequences. These studies provide the first proof of principle that combining genetic correction in hiPSCs will generate clinically relevant cells for autologous cell-based therapies. 

Wilson’s disease (WD) is an autosomal recessive inborn error of copper metabolism. Mutations in the ATP7B gene (located in chromosome 13) are responsible for WD with a prevalence of 1 in 30,000–100,000 [33]. Zhang *et al*. [16] described the generation of iPSCs from a Chinese patient with WD bearing the R778L “Chinese hotspot” mutation in the ATP7B gene. These iPSCs were pluripotent and could be readily differentiated into HLCs that displayed abnormal cytoplasmic localization of mutated ATP7B and defective copper transport. This phenomenon is susceptible to correction using a chaperone drug. Gene correction was performed in HLCs using a self-inactivating lentiviral vector that expresses codon optimized-ATP7B. The newly produced HLCs reversed the functional defect *in vitro*. Hence, their work describes an attractive model for studying the pathogenesis of WD that is valuable for screening compounds or gene therapy approaches aimed to correct the abnormality. This approach may be used for other diagnosis and correction of diseases susceptible to gene therapy. Genetically corrected, characterized lines of patient-specific iPSCs can be obtained in 4–5 months [34].

### 3.4. iPSCs in Liver Diseases Model

Liver tissue from patients is difficult to obtain and only reveals the disease aftermath, so several genetic disorders have been modeled in rodents and large animals [7]. Although these models of human inherited metabolic disease are invaluable, they provide a limited representation of human pathophysiology [35]. Animal models, especially those in rodents, do not always faithfully mimic human diseases, and most are imperfect [7]. Therefore, new advances in experimental techniques are needed to develop new models of human liver disease, especially large animal models that may be of greater clinical relevance [36]. Transplantation of hiPSCs into immunodeficient pigs with formation of humanized xenografts offers great potential [37].

#### 3.4.1. iPSCs from Normal Individuals

Disease modelling and drug screening are two immediate applications of the reprogramming technology and the resulting iPSCs differentiated cells. hiPSCs offer the ability to produce host-specific differentiated cells and thus have the potential to transform the study of infectious disease. HLCs derived from hiPSCs are particularly important for patients with liver diseases who cannot undergo surgical biopsy for the isolation of hepatocytes for transplantation. Disease modelling using iPSCs has been achieved for a variety of genetic diseases [38]. 

Research on HBV or HCV has been hampered by difficulty in culturing human primary hepatocytes, which tend to dedifferentiate and lose hepatic function after a limited time *in vitro*. Thus, alternative models have been used. *In vitro* models using animal hepatocytes, human HCC cell lines, or *in vivo* transgenic mouse models have contributed to understanding the pathogenesis of HBV and HCV [17]. However, host tropism of HBV or HCV is limited to human and chimpanzee. HBV and HCV infection has never been fully understood because there are few conventional models for hepatotropic virus infection. hiPSCs-derived HLCs from normal individuals would be useful for modelling susceptibility to infectious diseases. These hybrid cells provide an opportunity to elucidate the genetic basis of the mechanisms underlying cell susceptibility or resistance to viruses. In particular, HLCs derived from iPSCs of normal subjects are an appropriate target for studying the interactions between the host and virus with hepatic tropism. 

HCV is a prototypic pathogen for which host genetic factors have been implicated in modulating disease natural history and treatment response but whose functions remain poorly understood because of the lack of robust experimental systems. Yoshida [18] group investigated the entry and genomic replication of HCV in iPSCs-derived HLCs by using HCV pseudotype virus (HCVpv) and HCV subgenomic replicons, respectively. They showed that iPSCs-derived HLCs, but not iPSCs, were susceptible to infection with HCVpv. The iPSCs-derived HLCs expressed HCV receptors. HCV RNA genome replication occurred in the iPSCs-derived HLCs. Anti-CD81 antibody, an inhibitor of HCV entry, and interferon, an inhibitor of HCV genomic replication, dose-dependently attenuated HCVpv entry and HCV subgenomic replication in iPSCs-derived HLCs, respectively. These findings suggest that iPSCs-derived HLCs are suitable *in vitro* models of hepatocytes for the study of HCV infection. Schwartz reported that hiPSC-derived HLCs support the entire life cycle of HCV [19], including inflammatory responses to infection, enabling studies of how host genetics impact viral pathogenesis. Such models will advance our understanding of host–pathogen interactions and help realize the potential of personalized medicine.

#### 3.4.2. iPSCs from Patients

Monogenic metabolic disorders of the liver are an ideal platform to explore the complexity of gene–environment interactions and the role of genetic variation in the onset and progression of liver disease. The use of human hepatocyte cultures may circumvent the problems of animal models of human diseases in some sense. Many traditional cell-based models have been used to study pathogenesis and to screen for candidate drugs. However, none has used symptom-relevant human cell types since these cells are difficult to obtain, and under monolayer culture conditions hepatocytes lose their liver specific functions within a few days. Disease-relevant cell types could accurately reflect disease pathogenesis *in vitro*. iPSCs generated from patients who have monogenic inherited liver diseases and HLCs derived from iPSCs can be used as instruments to study the pathogenesis, disease mechanism(s) and possible cures for inherited liver disorders.

Current animal models of WD, including the toxic milk mouse, ATP7B2/2 mouse and Long-Evans Cinnamon rat, have provided very useful representation concerning its pathogenesis. However, physiological differences in phenotype between species limit the conclusions. As mentioned earlier, Zhang reported establishment of an *in vitro* disease model using iPSCs from WD patients [16]. 

Recently, several liver-specific disease iPSCs, such as familial hypercholesterolemia (FH), glycogen storage diseases (GSD), Crigler-Najjar syndrome, A1AT deficiency and FH have been launched [5,20]. These cells can be used as suitable specific models to study the pathogenesis, mechanism(s) and possible treatment for inherited liver disorders.

Rashid *et al*. demonstrate the possibility of modeling groups of diseases whose phenotypes are a consequence of complex protein dysregulation within adult cells. They derived iPSCs from the skin fibroblasts of patients with A1AT deficiency, GSD type 1a, FH, Crigler-Najjar syndrome type 1 and hereditary tyrosinemia. These iPSCs were then differentiated into HLCs, and characterized with special attention to the phenotypic properties specific to the corresponding diseases [5]. Rashid *et al*.’s results demonstrated that hiPSCs-derived HLCs can be generated from multiple patients of varied genetic and disease backgrounds. Their system has proved to be an efficient methodology for screening of early-stage safety and therapeutic effect of liver-targeted compounds of potential relevance to the pharmaceutical industry. 

Ghodsizadeh [20] derived iPSCs from liver-specific patients with tyrosinemia, GSD, progressive familial hereditary cholestasis, and two siblings with Crigler-Najjar syndrome. The hepatic lineage-directed differentiation of the iPSCs showed that the HLCs expressed hepatocyte-specific markers. Functionality of these cells was confirmed by glycogen storage and lipid storage activity, secretion of albumin, alpha-fetoprotein, and urea, CYP metabolic activity, as well as LDL and indocyanine green uptake. The large array of iPSCs lines produced in these studies will permit more in-depth characterization of disease phenotypes. The patient-derived HLCs from iPSCs can also be used as suitable specific models to study the pathogenesis, mechanism(s) and possible treatment for inherited liver disorders.

### 3.5. In Drug Discovery and Hepatoxicity Screening

An added benefit of iPSCs is that they can be used for drug screening. Adverse drug reactions continue to pose a major problem to the clinician, the pharmaceutical industry and the regulatory authorities. Amongst the different types of adverse drug reactions, drug-induced liver injury (DILI) is the most prominent cause of patient morbidity and mortality. Thus, a multitude of new drugs need to be efficiently screened every year to assess their potential for toxicity. A major challenge for drug discovery is to develop appropriate preclinical models. Human primary hepatocytes have become a major liver model for hepatotoxicity tests. Unfortunately, as mentioned above, there is also a shortage of primary hepatocytes, and it is difficult to culture the hepatocytes *in vivo* without losing their depth and breadth of specialized functions, and their limited availability, inter-donor differences, variable viability following isolation and rapid dedifferentiation of the hepatocyte phenotype in culture, particularly in the loss of CYP enzyme expression, impede their use. The pluripotent nature and the indefinite proliferative potential of ESCs are two major detractions of using ESCs in safety research in the fields of pharmacology and toxicology. However, directed differentiation of human ESCs to mature hepatocyte phenotypes *in vitro* could provide a readily available source of hepatocytes for early stage safety testing. Today, approximately 70% of the top 20 pharmaceutical companies utilize stem cells in their research and among these, 64% use human ESCs or their derivatives. Human ESCs and their derivatives do not encompass all the variances within a population or between ethnicities. Alternatively, ideal cells for drug screening could be obtained from iPSCs-derived HLCs. Since cells from patients with many different metabolism phenotypes must be tested to establish safety, hiPSCs-derived HLCs from this wide range of patients are expected to improve the drug discovery process [5,21] and may lead to personalized drug administration. Specifically, iPSC-hepatocytes generated from individuals with different CYP polymorphisms would be of great value for study of drug metabolism and toxicity prediction of new drugs [7]. Moreover, iPSCs offer the opportunity to generate liver cells at different stages of maturation, as well as the potential to give rise to all the composite cells of the adult liver, which may provide extra advantages and substantially expand the scope of traditional studies in drug metabolism and toxicology [22]. Choi *et al*. used patient-specific iPSCs, screened the clinical-ready drug library (the JHDL), and identified and validated several hits for novel treatment of A1AT deficiency. With emerging new tools and technologies for gene manipulation, such as transcription activator-like effector nucleases (TALENs) and clustered regularly interspaced short palindromic repeats (CRISPRs), the feasibility of iPSC-based large-scale drug screening and highly efficient gene correction are anticipated. Integration of patient-specific iPSC-based screening in early stages of drug development will help to more accurately predict drug effects in humans, thereby significantly shortening the timeline and reducing the costs associated with clinical trials and high failure rates [23]. In view of the potential of hiPSCs in providing an alternative model for safety pharmacology and toxicology applications, many pharmaceutical and biotechnology companies in recent years have invested or have developed joint collaborations with academia, to develop *in vitro* systems based on hiPSCs [24]. Moreover, the potential to make genetically corrected hiPSCs from a diverse number of diseases and genetic subtypes also allows for the development of reliable models for studying the development and progression of genetic diseases *in vitro* [23,25]. For example, disease-causing gene mutation and/or correction of hiPSCs offer ideal controls for comparative studies of pharmaceutical agents *in vitro*.

## 4. Challenges of iPSCs Application

### 4.1. Large Expansion System of iPSCs

A major technical hurdle that must be overcome before iPSCs can be implemented clinically is scalability, referring to the reproducible production of cells and their differentiated progeny on a large scale. All of the iPSCs lines established thus far have been generated and expanded under static tissue culture protocols, which are time-consuming and suffer from batch-to-batch variability. Additionally, monolayer culture provides limited numbers of iPSCs, only sufficient for research. Therefore, large scale systems for rapid expansion and maintenance of iPSC’s and their differentiated progeny are required for further research as well as future clinical applications.

Shafa reported expansion and long-term maintenance of iPSCs in a stirred suspension bioreactor (SSB) [39]. Their study showed that murine iPSCs can be maintained and expanded in SSB without loss of pluripotency over a long-term period. Kehoe also reported scalable SSB culture of hiPSCs [40]. They demonstrated SSB cultured iPSCs as aggregates, and the iPSCs aggregates retained the ability to express pluripotency markers, as well as the potential for multi-lineage differentiation *in vitro* and *in vivo*. Chen described the use of microcarriers (MCs) in suspension culture bioreactors for iPSCs cultivation [41]. Such a 3-dimensional culture system represents an efficient process for the large-scale expansion and maintenance of iPSCs, which is an important first step in their clinical application.

### 4.2. Immaturity of the HLCs Derived from iPSCs

Prior to clinical application, HLCs derived from iPSCs must be compared to primary liver-derived cells and shown to have similar morphology and functional properties such as nutrient processing, detoxification, plasma protein synthesis, and engraftment after transplantation into a suitable animal model. While a wealth of studies highlight the promise of iPSC-derived HLCs for transplantation therapies, several obstacles remain. So far, neither ESCs nor iPSCs can differentiate to fully mature hepatocytes *in vitro*. Researchers have then termed such populations of cells derived from iPSCs or ESCs as hepatocyte-like cells or “HLCs”. HLCs indicates that only some of the properties of mature hepatocytes are present. In general, HLCs demonstrate lower rates of albumin production, incomplete urea cycle activity, lower CYP activity, immature mitochondria and lower oxygen consumption than primary hepatocytes [3]. HLCs also show persistent expression and high levels of AFP production, suggesting that HLCs exhibit an inability to turn off early stage gene(s) as the mechanism of persistent immature phenotype [42]. Moreover, despite recent advances, the efficiency of human ESCs and iPSCs directed-differentiation into HLCs is highly variable and cell line-dependent. Since the undifferentiated iPSCs have the potential to form teratoma, research must be actively pursued to gain more information in order to clearly delineate the differentiation pathways of iPSC into specific cell types to ensure similar function and physiology. 

To address the issue of maturation of HLCs from iPSCs, Ogawa [43] used a method of modified growth factor and a 32-day 3D differentiation to show that the combination of 3D cell aggregation and cAMP signaling enhanced the maturation of hiPSCs-derived hepatoblasts to a hepatocyte-like population. The resulting cells displayed expression profiles and metabolic enzyme levels comparable to those of primary human hepatocytes. Importantly, they also demonstrated that generation of the hepatoblast population capable of responding to cAMP is dependent on appropriate activin/nodal signaling in the definitive endoderm at early stages of differentiation. Together, these findings provide new insights into the pathways that regulate maturation of iPSCs-derived HLCs. In doing so, they provide a simple and reproducible approach for generating metabolically functional hepatocytes.

Shan [44] used a screening approach involving two different classes of small molecules to identify factors that induce the proliferation of mature primary human hepatocytes or induce the maturation of HLCs from hiPSCs. The first class induced functional proliferation of primary human hepatocytes *in vitro*. The second class enhanced hepatocyte functions and promoted the differentiation of iPSCs-derived HLCs toward a more mature phenotype than what was previously obtainable. Gene expression profiles showed that HLCs treated with small molecules more closely resembled mature hepatocytes. Marked increases in the amount of albumin and CYP3A were seen with treated cells *vs*. untreated cells. Of particular interest, AFP was largely absent in treated cells. The identification of these small molecules may have an impact on several areas of research, including maturation of other iPSCs-derived cell types, expansion of other “terminally” differentiated cell types, and the translational potential of these cell types. 

Zhang *et al*. [8] directly compared the hepatic-differentiation capacity of mouse iPSCs with three different induction approaches: conditions via embryonic body formation plus cytokines, conditions by combination of DMSO, and sodium butyrate, and chemically defined N2B27 medium, serum free monolayer conditions. In the mid-term induction stage, the investigators added sodium butyrate, a short-chain fatty acid and a histone deacetylase inhibitor. Sodium butyrate has been reported to induce growth arrest, differentiation, and apoptosis of cancer cells in chemically defined, serum free medium. Among these three induction conditions, more homogenous populations can be promoted under serum free conditions. Although efficient hepatic differentiation was achieved by these modifications, the present protocols are far from perfect. Further optimization is needed for clinical application of iPSCs-derived HLCs. Efforts are underway to define an ideal hepatic induction strategy for future individualized hepatocyte transplantation.

The induction of HLCs from iPSCs is a complicated process that will eventually be replaced by less complex technology. Huang *et al*. demonstrated the direct induction of functional HLCs (named as iHep cells) from mouse tail-tip fibroblasts. Direct induction was accomplished by single step transduction of Gata4, Hnf1a and Foxa3, and inactivation of p19^Arf^. iHep cells show typical epithelial morphology, express hepatic genes, and perform hepatocyte functions. Notably, iHep cells showed an expression profile and hepatic function very similar to mature hepatocytes. Donor iHep cells repopulate the livers of FAH-deficient mice and rescued almost half of recipients from death by restoring liver functions. More importantly, iHep cells did not form tumors in immunodeficient mice. Their study provides a novel strategy to generate functional HLCs for the purpose of liver regenerative medicine [45].

### 4.3. Strategies to Purify the HCLs Differentiated from iPSCs

Under current situations, transplantation of differentiated iPSCs into patients is risky as the residual undifferentiated iPSCs may retain the possibility of tumor formation. Therefore, the safety of clinical cell transplantation using differentiated hiPSC derivatives is contingent on novel methods to remove the undifferentiated iPSCs [46]. To date, strategies for purifying a given cell population have used either a cell surface protein specific for the target cell population, such as a cell surface marker specific to hepatic progenitors, or lentivectors expressing a reporter gene under the control of a specific promoter [47]. For example, to purify HLCs from a heterogeneic population, elegant experiments by Basma *et al*. [48] generated human HLCs through embryoid bodies. These cell aggregates were purified using fluorescence-activated cell sorting for the asialoglycoprotein receptor. Purified epithelial cell adhesion molecule EpCAM-positive cells from fetal and postnatal livers have also been used to generate mature hepatocytes [49]. The EpCAM marker is also expressed in the visceral endoderm and in several progenitor cell populations and cancers, and is associated with undifferentiated hESCs [50,51]. Therefore, to date, purification of progenitors and mature cells generated from either ESCs or iPSCs remains challenging with use of conventional methods. More studies need to be carried out to develop better purification methods before iPSC can be used clinically. 

Yang *et al*. [52] reported the use of lentivectors encoding green fluorescent protein (GFP) driven by the liver-specific apoliprotein A-II (APOA-II) promoter to purify human hepatic progenitors. The investigators first differentiated a human ESC line into hepatic progenitors using a chemically defined protocol. Subsequently, cells were transduced with GFP and sorted at day 16 of differentiation to obtain a cell population enriched in hepatic progenitor cells. After sorting, more than 99% of these APOA-II-GFP-positive cells expressed hepatoblast markers such as AFP and cytokeratin 19. When cultured for an additional 16 days, the sorted hepatoblasts underwent differentiation into more mature cells and exhibited hepatocyte properties such as albumin secretion. Moreover, they were devoid of viral DNA integration. Their strategy produces a novel tool that could be used not only for cell therapy but also for *in vitro* applications such as drug screening. The present strategy should also be suitable for the purification of a broad range of cell types derived from either iPSCs or adult stem cells.

### 4.4. Low Efficiency of Engraftment

Functionality of human cells differentiated *in vitro* is currently best tested by transplantation into immunodeficient rodent models. However, low efficiency of engraftment and proliferation of transplanted cells into the host parenchyma is a limitation that must be considered. Alternatively, a selective growth advantage of donor cells over endogenous cells may address this limitation. For example, in some models, the survival and/or proliferation of native hepatocytes is impaired by a genetic or inherited inability to regenerate, as in fumarylacetoacetate hydrolase (FAH)-deficient mice and urokinase (Alb-uPA) transgenic mice [53,54]. These two murine models have been crossed with immunodeficient mice with a different genetic background [1]. Even with a suitable animal model, human HLCs generated from pluripotent or multipotent stem cells currently repopulate transplanted livers less efficiently than primary human hepatocytes [55]. These results suggest that fully mature donor HLCs may achieve higher engraftment efficiency.

### 4.5. EP Cell Lines from iPSCs

Besides the high variability and efficiency of differentiation, the pluripotent nature of ESCs and iPSCs results in production of cells types from different germ layers in most differentiation protocols. Thus, it is difficult to produce pure monolineage cultures of a desired cell type from iPSCs [56]. An effective method to direct iPSCs differentiation is to use an established definitive germ layer stem cell line, such as a definitive endoderm (DE) progenitor line. The spectrum of differentiation of definitive germ layer stem cells is relatively narrow. Thus, the efficiency of directional differentiation from definitive germ layer stem cells to a specific cell type can be increased. On the other hand, definitive germ layer stem cells have broader differentiation potential than tissue stem cells, which is economical. Furthermore, definitive germ layer stem cell lines are less tumorigenic than ESCs and iPSCs. Endoderm stem cells can differentiate to the liver, pancreas, intestines, stomach, lung and other organs cells, but not teratomas. Therefore, endoderm stem cells have potential in clinical application.

As noted earlier, differentiation from iPSCs toward hepatic lineage cells mimics *in vivo* step-wise developmental processes. Therefore, hiPSCs-derived hepatic progenitor-like cells (HPCs) might exist at an appropriate time point during similar *in vitro* differentiation steps. Yanagida reported that after differentiating with defined cytokines, HPCs from hiPSCs can be highly purified using cell surface markers CD13 and CD133. Further investigation revealed that hiPSCs-derived HPCs exhibit a long-term proliferative potential and maintain bipotent differentiation toward hepatocytic cells and cholangiocytic cells [57]. Their human HPCs derived from iPSCs may be useful for the analysis of human hepatic cell development. In addition, mature hepatocytes lose proliferative ability after cryopreservation. In contrast, hiPSCs-derived HPCs have a highly proliferate ability even after cryopreservation. Thus, the *in vitro* expansion system of HPCs may contribute to regenerative therapies of liver diseases using functional human hepatic progenitor cells and hepatocytes. 

Recently, Cheng *et al*. [58] generated self-renewing DE progenitor lines from both human ESCs and iPSCs. These cells, termed endodermal progenitor (EP) cell lines, displayed a proliferative capacity similar to ESCs, yet lacked teratoma-forming ability. In addition, EP cell lines generated endodermal tissues representing liver, pancreas, and intestine, both *in vitro* and *in vivo*. EP cell lines provide a powerful reagent to study gut tissues from a common multipotent endodermal progenitor and to optimize monolineage differentiation. Moreover, creation of EP cells from ESCs/iPSCs may represent a strategy to optimize the production of pure, non-tumorigenic cells for tissue replacement therapies.

### 4.6. Large Expansion System of HLCs and Engineering Liver with iPSCs

Large scale production of HLCs is needed for their clinical application. As mentioned earlier, generation of HLCs from iPSCs is very time consuming under monolayer culture conditions. Vosough [59] reported their generation of functional HLCs from hiPSCs in a scalable suspension culture with rapamycin for “priming” and activin A for induction. After transplantation of these HLCs into the spleens of mice with acute liver injury, an increased rate of survival was observed. Improved survival correlated with cell engraftment in the liver and hepatic function (*i.e*., albumin secretion after implantation). This novel enrichment strategy provides a new platform for generating HLCs, and it may open new windows in the clinical and pharmaceutical application of these cells.

It has been shown that the efficient function of multiple cell types, including hepatocytes and islet hormone-producing cells, is dependent on matrix-producing cells and endothelial cells that provide a 3D support structure and sufficient vascularization [60,61,62]. Thus, the liver extracellular matrix presents an ideal scaffold for stem-cell differentiation into hepatocytes [63,64]. It is known that local environmental factors induce hepatocyte homing, differentiation, and proliferation, and studies indicate that stem cells may differentiate toward mature hepatocytes following transfer into an injured liver. Therefore, the decellularized liver matrix has significant potential as the scaffold for hepatocyte maturation. This process may be further promoted by the sequential delivery of factors involved in the initiation and maturation of stem cells to liver cells [48], allowing temporal and spatial control over differentiation. Hannan [65] described a 25-day protocol to direct the differentiation of human pluripotent stem cells into a near homogenous population of HLCs. They demonstrated that day 25 of this protocol represents the earliest time point at which cells can be used to model basic hepatic metabolic function. However, cells differentiated at day 35 systematically displayed the highest level of albumin secretion and CYP activity, suggesting that the later date was optimal for functional analyses and toxicology screening. This delayed approach enables the generation of a larger quantities of hESCs/hiPSCs for differentiation into hepatocyte-like cells and their clinical applications.

### 4.7. In Vivo Differentiation of iPSCs

Because even a small number of undifferentiated cells can result in teratoma formation, a goal of iPSCs differentiation is to avoid production of undifferentiated cells. To date, no iPSCs-derived differentiation protocol has succeeded in yielding high purity HLCs that fulfill both functional engraftment and response to proliferative stimuli in the diseased liver. Alternative strategies are needed to obtain mature hepatocytes. To exclude compensation by hepatocytes not derived from iPSCs, Espejel *et al*. transferred wild-type mouse iPSCs into the embryos of FAH-deficient mice to generate chimeric mice. These mice demonstrated the ability of iPSCs to develop into hepatocytes *in vivo*. Furthermore, recipient FAH-deficient mice were protected from developing hepatic failure [11]. Zhao also produced mice using iPSCs and tetraploid complementation [66], which can provide the liver organ for engineering liver. The tumor formation potential of these cells has not been completely eliminated. 

Takebe and his team grew bioengineered liver tissue from hiPSCs by reprogramming human skin cells to an embryo-like state. The researchers first placed the iPSCs on growth plates in a custom medium. After nine days, the mature cells were characterized by biochemical markers as hepatocytes. Umbilical endothelial cells and mesenchymal cells were then added to the culture system to induce formation of blood vessels and stroma, respectively. Two days later, 3D tissues of 5 mm width were observed, which the researchers described as a “liver bud” at an early stage of liver development [67]. The liver-buds were transplanted and examined histologically at multiple time points. Of the cells from the hiPSC-derived liver buds, 32.9% are albumin positive. These buds quickly attached to nearby blood vessels and grew rigorously after transplantation. The vascular networks of liver buds were similar in density and morphology to those of adult livers after transplantation. 

Chan reported that they directly transplanted iPSCs into CCl_4_-induced liver injured mice [68]. They found that mice with transplants of iPSCs performed better than mice with transplants of iPSCs-derived HLCs. Performance was assessed by levels of serum alanine aminotransferase, aspartate aminotransferase, and liver necrosis. The protective effects of iPSCs were associated with increased chemokine inducible protein 10 (IP-10), a potential regulatory factor for amelioration of liver injury *in vivo*. 

Other possibilities for large scale expansion and maturation of HLCs include a genetically engineered large animal model [36] to serve as an *in vivo* hepatocyte incubator [69]. Prior studies have established immunodeficient FAH−/− mice for this purpose [55]. Exposure to damaged liver tissue stimulates liver cell regeneration and can enhance homing and differentiation of stem cells to a hepatocyte phenotype. The future success of *ex vivo* cell therapies depends on novel techniques to provide an abundant, high quality supply of functionally normal hepatocytes.

## 5. Conclusions

iPSCs present exciting possibilities for the study and treatment of liver diseases. Areas of study and treatment include *in vitro* modeling, *in vivo* modeling of diseases, drug development, tissue engineering, and development of BAL devices. iPSCs also provide novel opportunities for autologous cell therapies and cell transplantation without risk of immune rejection. However, there are still several obstacles that need to be overcome before iPSCs reach the bedside. These include: (i) improved efficiency of iPSCs generation without viral integration; (ii) avoidance of animal feeders to culture hiPSCs; (iii) novel differentiation protocols for more efficient and economical production of mature cell types whose functionality are comparable to their *in vivo* counterparts; (iv) rapid differentiation protocols for emergent usage; and (v) enrichment of desired (mature) cells and removal of undesired (undifferentiated) cell types that have the potential for tumor formation *in vivo*. A recent report [70] that undifferentiated iPSCs elicit T-cell-dependent immune responses in syngeneic mice will require further investigation. This report suggests that host immune responses may be important for the removal of undifferentiated cells due to their abnormal expression of antigens following genetic manipulation.

A thorough preclinical assessment of iPSCs in suitable large-animal models is prudent to ensure that the proposed treatment with iPSC-derived cells is both safe and effective before testing in humans. It has reported recently that transplantation of undifferentiated iPSCs demonstrated T-cell-dependent immune response in recipient syngeneic mice due to the abnormal expression of antigens following genetic manipulation [70]. Therefore, critical aspects need to be further addressed, including the long-term safety, tolerability, and efficacy of the iPSC-based treatments. It is paramount to conduct well-designed clinical trials to fully establish the safety profile of such therapies and to define the target patient groups with efficacy assessed by standardized protocols. Despite their limitations, iPSC-derived hepatocytes remain a promising population for liver cell therapies. Moreover, engineered donor grafts derived from iPSCs, including re-cellularized biomatrix [71] and liver buds produced from iPSCs [67], may someday provide organs for liver transplantation. These results highlight the enormous therapeutic potential for treating organ failure.

## Abbreviations

A1AT: alpha-1-antitrypsin
ALF: acute liver failure
APOA-II: apoliprotein A-II
BAL: bioartificial liver
CYP: cytochrome P450
DMSO: dimethyl sulfoxide
DE: definitive endoderm
EP: endodermal progenitor
ESCs: embryonic stem cells
FAH: fumarylacetoacetate hydrolase
hiPSCs: human iPSCs
HNF4α: hepatic nuclear factor 4-alpha
HPCs: hepatic progenitor-like cells
iPSCs: induced pluripotent stem cells
HLC: hepatocyte-like cells
WD: Wilson’s disease
